# Changes in the Immune Cell Repertoire for the Treatment of Malignant Melanoma

**DOI:** 10.3390/ijms232112991

**Published:** 2022-10-27

**Authors:** Kenta Nakamura, Ryuhei Okuyama

**Affiliations:** Department of Dermatology, Shinshu University School of Medicine, Matsumoto 390-8621, Japan

**Keywords:** melanoma, immune checkpoint inhibitor, repertoire, TCR, BCR

## Abstract

Immune checkpoint inhibitors (ICIs) have been used for the treatment of various types of cancers, including malignant melanoma. Mechanistic exploration of tumor immune responses is essential to improve the therapeutic efficacy of ICIs. Since tumor immune responses are based on antigen-specific immune responses, investigators have focused on T cell receptors (TCRs) and have analyzed changes in the TCR repertoire. The proliferation of T cell clones against tumor antigens is detected in patients who respond to treatment with ICIs. The proliferation of these T cell clones is observed within tumors as well as in the peripheral blood. Clonal proliferation has been detected not only in CD8-positive T cells but also in CD4-positive T cells, resident memory T cells, and B cells. Moreover, changes in the repertoire at an early stage of treatment seem to be useful for predicting the therapeutic efficacy of ICIs. Further analyses of the repertoire of immune cells are desirable to improve and predict the therapeutic efficacy of ICIs.

## 1. Introduction

Immune checkpoint inhibitors (ICIs) are currently used for the treatment of various types of cancers, including malignant melanoma [1]. However, only approximately 30–50% of melanoma patients respond to treatment with ICIs; thus, their therapeutic efficacy needs to be improved [2,3]. The analysis of “tumor antigen-specific immune responses”, which represent an important part of immune responses to tumors, can aid in improving the therapeutic efficacy of ICIs [4,5]. Therefore, changes in the repertoires of immune cells that recognize tumor antigens have been analyzed [6]. Multiple studies have reported that the ICI-induced restoration of exhausted antitumor immune responses allows for the clonal proliferation of immune cells and produces therapeutic benefits [7,8,9]. In this article, we review recent progression on the repertoires of various immune cells and their associations with treatment and symbiotic bacteria in malignant melanoma.

## 2. T Cells and Their Repertoires

### 2.1. T Cell Receptor (TCR)

T cells are activated by the recognition of corresponding antigens via TCRs. Most T cells express αβTCR, a heterodimer of TCRα and TCRβ chains, whereas some T cells express γδTCR, a heterodimer of TCRγ and TCRδ chains. T cells expressing αβTCR are referred to as αβT cells, whereas T cells expressing γδTCR are referred to as γδT cells.

There are between 2 × 10^9^ and 6 × 10^9^ unique TCRs in the circulating T cell population of a healthy individual [10]. Such a large number of unique clonotypes are stochastically generated via V(D)J recombination in thymic T cell progenitors, followed by positive and negative selection, which select T cells that bind to major histocompatibility complex (MHC) but not to self-peptides. TCRs are responsible for the sensitivity and specificity of T cells to peptides presented by MHC [11]. Once the TCR binds to a peptide, signal transduction occurs, which promotes activation, proliferation, differentiation, cytokine production, and an increase in cytolytic activity [12]. Through unique TCRs on individual T cells, T cells can scan antigens presented by MHC molecules on the surface of tumor cells [13]. The recognition of an antigen by a TCR results in the activation and proliferation of antigen-reactive T cells, and a process of clonal proliferation is triggered [14].

Tumor-specific T cells develop as a response to tumor antigens, including individual “neoantigens” derived from mutant proteins of cancer cells [15]. The large variety of TCRs required for effective immune function is produced via error-prone recombination of the TCR loci [16,17]. In particular, complementarity determining region 3 (CDR3) of the TCR is highly diversified and has sequences that are unique to individual T cell clones, and T cell responses can be traced by sequencing the CDR3 regions of TCRs. The sequencing efforts have been focused particularly on CD8-positive T cells, which play a central role in immune responses to tumors. The therapeutic efficacy of ICIs is correlated with the proliferation of T cell clones possessing TCRs that recognize tumor antigens (decrease in the T cell repertoire size) [7]. The therapeutic efficacy of ICIs and changes in the repertoire are described in detail later.

### 2.2. CD8-Positive T Cells

CD8-positive T cells recognize tumor antigens via MHC class I and play a major role in tumor immunity. Therefore, the tumor-infiltrating CD8-positive T cell count is a prognostic factor in patients with cancer [18].

Tumor-infiltrating lymphocytes (TILs) form a polyclonal population in which T cells targeting known and unknown tumor-specific antigens, such as tumor-associated antigens and neoantigens, are enriched [18]. A recent study showed a relatively large overlap between the TCR repertoire of TILs and that of CD8-positive PD-1 (programmed cell death 1)-positive T cells in the peripheral blood [19]. Furthermore, the TCR repertoire of peripheral blood PD-1-positive lymphocytes proliferated in vitro is highly similar to that of TILs [20]. Treatment with these proliferated lymphocytes administered concomitantly with an anti-PD-1 antibody led to successful outcomes in melanoma patients [7].

Furthermore, the TCR repertoire in melanoma patients before starting immunotherapy was compared with that in healthy individuals [21]. The TCR repertoire of CD8-positive T cells in the blood of melanoma patients was significantly more restricted than that in healthy individuals. In melanoma patients, the repertoire of CD8-positive T cells in the peripheral blood was more restricted than that of CD4-positive T cells. In addition, the same CD8-positive T cell clones were enriched in the peripheral blood and tumors after treatment with an anti-CTLA-4 (cytotoxic T-lymphocyte-associated protein 4) antibody. In another study, T cell clones present in both tumors and peripheral blood were investigated to analyze their ongoing immune responses [22].

A single-cell level analysis of transcriptome and TCRαβ repertoire pairs was conducted with peripheral blood T cells and tumor-infiltrating T cells from matched patients with metastatic melanoma. The effector function gene signatures in clonally proliferated TILs were similar to those in T cells in matched peripheral blood. In contrast, exhaustion factors (e.g., PD-1) were expressed mainly on tumor-infiltrating T cells. Moreover, significantly more peripheral blood T cells possessing a tumor antigen-recognizing repertoire were found in tumor tissues after immunotherapy. These data suggest that peripheral blood T cells are useful for assessing T cell function in cancer immunotherapy.

Interestingly, clinical responses to immunotherapy are associated with an influx of novel T cell clones from the peripheral blood into the tumor microenvironment [8]. In other words, peripheral blood T cells are supplied as TILs into the tumor and are involved in tumor rejection. T cells primed locally in tumors are more likely to express exhaustion factors (e.g., PD-1) than peripheral blood T cells. In fact, some antigen-presenting cells (APCs) are unsuitable for priming T cell responses in tumors as a result of interactions with regulatory (Treg) T cells [23].

### 2.3. CD4-Positive T Cells

CD4-positive T cells recognize tumor antigens via MHC class II and assist CD8-positive T cells in antitumor immunity [24]. Maintenance of CD4-positive T cells depends on the presence of APCs, which include B cells, macrophages, and dendritic cells (DCs). CD4-positive T cells typically do not have a cytotoxic function; however, their role in cancer has been revisited recently owing to the fact they are required for proper activation of immune responses and maintenance of immunological memory [25]. The pretreatment TCR repertoire of CD4-positive T cells in melanoma patients is significantly more restricted than that in healthy individuals [22]. Moreover, CD4-positive T cell clones in peripheral blood proliferate in melanoma patients after treatment with an anti-CTLA-4 antibody, and these CD4-positive T cell clones are enriched in corresponding tumors. In addition, CD4-positive T cell clones are less proliferative than CD8-positive T cell clones [26]. Investigation of tertiary lymphoid structures in human cancer may be a feasible approach to analyze the immune responses of CD4-positive T cells.

### 2.4. Regulatory T Cells

CD4-positive Treg cells play important roles in the maintenance of self-tolerance [27]. However, they play a negative role in antitumor immune responses [28]. In contrast to Treg cells circulating in the blood, Treg cells present in tumors have a profile of activated cells [29]. Increases in the number of Treg cells in tumors are correlated with disease progression and decreased survival in patients with cancer. However, the antigen specificity of Treg cells that undergo proliferation in the tumor microenvironment has not been fully understood. Thus, the TCR profiles of intratumoral Treg cells were analyzed to elucidate their antigen specificity [30]. The TCR repertoire of Treg cells in tumors varied but had significant overlap with that of Treg cells in peripheral blood. In contrast, the TCR repertoire of Treg cells had no overlap with that of conventional T cells in tumors or peripheral blood. The TCRs of Treg cells seem to have specific reactivity to tumor antigens and neoantigens. These findings suggest that intratumoral Treg cells affect the activation and clonal proliferation of T cells in a tumor antigen-selective manner. In addition, tumor antigen-specific Treg-derived TCRs have been detected in tumors and peripheral blood, suggesting that Treg cells in peripheral blood serve as a source of tumor-specific TCRs.

### 2.5. Effector Memory T (T_EM_) Cells, Central Memory T (T_cM_) Cells, and Resident Memory T (T_RM_) Cells

While recent research has focused on T cell responses to cancer immunotherapy, long-term immunological memory has not been analyzed fully. In general, some effector T cells differentiate into central memory T (T_CM_) cells and effector memory T (T_EM_) cells. T_CM_ cells express CCR7 and CD62L and are found in secondary lymphoid tissues. T_EM_ cells circulate in secondary lymphoid and peripheral tissues. T_EM_ and T_CM_ cells are chiefly found among CD8-positive memory T cells in the tumor microenvironment [31].

PD-1, an immune checkpoint protein, is expressed in effector, effector memory, and central memory CD8-positive T cells in addition to exhausted T cells [32]. Among TILs, the presence of T_CM_ cells serves as a positive predictor of response to immunotherapy [33]. This may be because T_CM_ cells are capable of expanding during ICI therapy.

T_CM_ cells are characterized by the transcription factors TCF7 and T-bet. These are essential for central memory and maintenance of more differentiated T cells, respectively. An analysis conducted in melanoma patients showed that T-bet and TCF7 are upregulated by BRAF/MEK inhibition [34]. The repertoire of T cells found in tumors before treatment is expanded after the administration of BRAF/MEK inhibitors. TCF7 is involved in the self-renewal and persistence of CD8-positive memory T cells [35]. T-bet is important to maintain the balance between T cell memory and effector differentiation pathways and to trigger Th1 responses characterized by the interferon gamma (IFN-γ) signature [36]. These findings suggest that TCF7 and T-bet induced during BRAF/MEK inhibition promote the repertoire expansion of intratumoral T cells [34].

Recently, resident memory T (T_RM_) cells have been discovered as a subset of long-lived memory T cells. T_RM_ cells express surface markers such as CD103 and reside in peripheral nonlymphoid tissues [37,38]. They play a role in immune responses to microbial infection in barrier tissues, such as the skin. It is likely that the presence of T_RM_ cells is associated with improved long-term prognosis in various types of cancers, including melanoma [39,40,41]. T_RM_ cells have been detected in tumors before treatment [42].

In addition, memory CD8-positive T cell responses were investigated in melanoma patients who showed a long-term response (for at least 1 year) to immunotherapy [43]. Single-cell RNA sequencing revealed T_RM_ cells that were shared between tumors and the vitiliginous skin. TCR sequence analysis identified clonotypes that coexisted as T_RM_ cells in the skin and as T_EM_ cells in the blood, and clonotypes found in tumors, skin, and peripheral blood expressed high levels of IFNγ and TNFα. Higher levels of these cells were strong predictors of favorable prognosis in melanoma patients. Intratumoral clonotypes were found in the skin and blood of patients for up to 9 years, and the tumor-related clone repertoire was abundantly maintained in the skin. In the future, analyses of the effects of T_RM_ and T_EM_ cells on the clonal proliferation of T cells by immunotherapy are anticipated.

## 3. B Cells and Their Repertoires

### 3.1. B Cell Antigen Receptors (BCRs)

BCRs are expressed on the surface of B cells and bind directly to antigens. When two or more BCRs recognize a multivalent antigen, the BCRs becomes cross-linked and signal transduction ensues [44]. This results in the proliferation, differentiation, and activation of B cells. Antigens bound to the BCRs are taken up by the cells and degraded into peptides, which are then presented by MHC class II. The antigen-presenting B cells interact with T cells that are reactive to the same antigens, inducing the formation of a germinal center and differentiation of plasma cells. In addition, antigen-recognizing B cells do not require the help of T cells for proliferation and differentiation into plasma cells.

### 3.2. B Cells

In melanoma, tumor-associated B cells account for up to 33% of immune cells [45]. Multiple studies on the role of tumor-associated B cells have been reported recently. The CD27-positive, CD38-positive, and PAX5-negative tumor-induced plasmablast-like-enriched B cell (TIPB) population regulates T cell-recruiting chemokines (CCL3, CCL4, and CCL5) and increases the number of tumor-infiltrating T cells. ICIs seem to be more effective in cases with a larger TIPB population [46], and tumor-associated B cells are related with improved survival independent of other clinical variables. This result may be explained by the induction of tertiary lymphoid structures [47,48]. Immunofluorescence staining of CD20, CXCR5, and CXCL13 revealed the formation of tertiary lymphoid structures in tumors. Interestingly, naïve B cells and plasma cells were more abundant in responders at baseline, and a higher number of intratumoral memory B cells was associated with a more diverse TCR repertoire. Furthermore, a higher number of plasma cells was associated with a higher number of infiltrating T cells. Among melanoma patients treated with ipilimumab and nivolumab, those with a larger number of immunoglobulin gene rearrangements in the tumors exhibited significantly longer progression free survival. Therefore, the intratumoral B cell repertoire should be evaluated to understand B cell immunity. The CDR3 sequences of B cell immunoglobulin heavy chain (IgH) and light chain (IgL) were assembled from bulk RNA-sequence data to analyze the function of intratumoral B cells in antitumor immune responses [49]. Clones of both IgH and IgL chains were significantly increased in number in tumors of patients who responded to treatment with ICIs. It is likely that the abundance of BCR clonotypes in tumors at baseline is positively correlated with the therapeutic efficacy of ICIs. The tumors of patients who responded to the treatment tend to have more clonal BCR repertoire.

## 4. Treatment and Repertoires

### 4.1. Changes in the Repertoire with ICIs

PD-1 blockade restores T cell functions primarily at the effector phase. Therefore, PD-1 blockade rescues exhausted CD8-positive T cells and restores their cytotoxic capacity, thereby facilitating the destruction of tumor cells [50]. Anti-PD-1 antibodies prevent PD-1 from binding to PD-L1 on APCs and interfering with the differentiation and functioning of Treg cells because PD-1 plays a role in the development of Treg cells [51]. However, the PD-1 expression pattern encompasses a broad range of immune cells, including T cells, B cells, natural killer cells, dendritic cells, and bone marrow cells. Its ligands, PD-L1 (programmed death-ligand 1) and PD-L2, are also expressed in various hematopoietic and nonhematopoietic cells as well as cancer cells [52,53]. Therefore, anti-PD-1 therapy can affect various types of cells and pathways.

Recent analyses have mainly focused on CD8-positive T cells, which play a major role in tumor immune responses. After anti-PD-1 therapy, most of the abundant TIL clones were found among T cell clones in peripheral blood, regardless of the clinical response [9]. T cell clones expressing TCRs that recognize tumor antigens proliferate in anti-PD1 antibody responders [7,54]. Moreover, an analysis in patients who underwent neoadjuvant anti-PD-1 antibody therapy revealed a post-treatment increase in the number of CD8-positive T cells reactive with gp100, a melanoma-specific antigen [55]. These findings suggest that the ICI-induced restoration of exhausted antitumor immune responses is followed by the clonal proliferation of immune cells, yielding therapeutic benefits.

**Figure 1:**
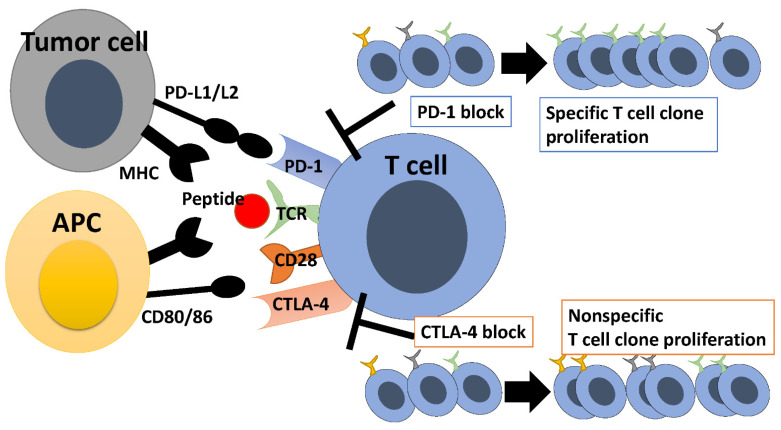
Repertoire changes with immune checkpoint inhibitors.

Meanwhile, blockade of CTLA-4, another immune checkpoint protein, decreases the T cell priming threshold and allows for the proliferation of more effector T cells [56]. Anti-CTLA-4 antibodies also allow for the proliferation of memory T cell clones [57]. Moreover, Treg cells in the tumor microenvironment are depleted as they express CTLA-4 [58]. Depletion of Treg cells improves intratumoral IL-2 levels, facilitates survival of CD8-positive T cells, and expands the TCR repertoire [59]. In other words, anti-CTLA-4 antibodies inhibit Treg cells, reverse inhibit CD8-positive T cells, and expand the TCR repertoire in a nonspecific manner [60]. In addition, anti-CTLA-4 antibodies accelerate the turnover of T cell repertoire and increase TCR diversity [61,62,63]. Anti-CTLA-4 antibodies broaden the T cell repertoire, whereas anti-PD1 antibodies promote the proliferation of a limited number of clones, skewing the T cell repertoire (Figure 1).

A previous study showed tumor antigenic changes after PD-1 blockade [64]. Thus, a dynamic clonal change in the TIL could be induced by antigenic alteration of tumor cells during treatment [64], whereas the clone of TIL expanded after PD-1 blockade responded to tumor cell line before PD-1 blockade [65]. Therefore, the authors reported that dynamic tumor-specific clonal changes after PD-1 blockade are caused by PD-1 blockade but not antigenic alteration of tumor cells [65].

T cells use TCRs to recognize antigen peptides presented by MHC on antigen-presenting cells. CD28 receives stimulus from CD80/86, which leads to T cell activation. CTLA-4 competes with CD28 and inhibits T cell activation. Therefore, inhibition of CTLA-4 results in nonspecific proliferation of T cell clones and expansion of the TCR repertoire. On the other hand, T cells use TCRs to recognize antigen peptides on tumor cells for eliciting antitumor immune responses, and tumor PD-L1 binding to PD-1 on T cells inhibits the antitumor immune responses. PD-1 inhibition promotes the proliferation of specific T cell clones, leading to alteration of the TCR repertoire.

### 4.2. Changes in the Repertoire with BRAF/MEK Inhibition

MEK inhibitors can impair T cell activation, because T cell activation mediated by TCRs and their co-stimulatory molecules is dependent on mitogen-activated protein kinase (MAPK) and the PI3K-AKT signaling cascade [66]. In fact, pharmacological in vitro inhibition of MEK had adverse effects on T cell activation [67].

In contrast, the results of an in vivo analysis were twofold: MEK inhibition had no adverse effects on T cell effector function and showed favorable outcomes in combination with immune checkpoint inhibition [68]. MEK inhibition was associated with increased tumor-infiltrating CD8-positive T cells, increased IFN-γ gene expression signatures, and decreased abundance of tumor-associated macrophages and Treg cells. Furthermore, MEK inhibition protected effector T cells from activation-induced cell death due to chronic TCR stimulation [69]. In addition, an analysis in melanoma patients showed that BRAF/MEK inhibition upregulated the levels of T-bet and TCF7 and expanded T cell repertoire in tumors [34]. Another study showed that higher intratumoral T cell clonality was associated with better responses to BRAF inhibitor treatment for melanoma [70]. Based on these results, efficacy analyses of ICIs combined with BRAF/MEK inhibitors are desired in clinical trials.

### 4.3. Changes in the Repertoire with Adoptive Cell Transfer of TIL Therapy

Adoptive cell transfer (ACT) of TIL is used for the treatment of advanced melanoma. ACT of TIL has shown significant clinical benefit [71]. However, ACT of TIL could not work enough in patients previously treated with PD-1 or MAPK inhibition [68]. Response to ACT correlates with the recognition of tumor neo-antigens [72,73]. Anti-PD-1 naïve patients were received TIL reactive with more neo-antigens compared with anti-PD-1 experienced patients [74]. Treatment products administered to anti-PD-1 naïve patients were more likely to contain T cells reactive against neoantigens than treatment products for anti-PD-1 experienced patients [74].

### 4.4. Changes in the Repertoire with IL-12 Therapy

IL-12, an inflammatory cytokine, induces the proliferation and activation of natural killer (NK) cells and cytotoxic T cells and enhances effector functions [75,76]. Additionally, it is an important link between innate and acquired immune systems, because APC-producing IL-12 stimulates the release of IFN-γ from T cells and NK cells [77]. It is also involved in Th1 induction and promotes IFN-γ production [78]. Thus, IL-12 plays a role in antitumor immunity, and T cells are important for IL-12-mediated tumor suppression [79]. Intratumoral plasmid IL-12 electroporation therapy was tested in a phase II trial in melanoma patients [80]. Following the treatment, intratumoral T cells proliferated clonally, which led to a skewed TCR repertoire.

### 4.5. Treatment Correlations and Repertoires

CTLA-4 is expressed mainly on CD4-positive T cells after TCR-mediated activation and interferes with CD28 co-stimulatory signaling induced by APCs to inhibit TCR-induced activation and proliferation [81]. Therefore, CTLA-4 inhibition induces the activation and proliferation of antitumor T cells via increased CD28 signaling.

PD-1 inhibits the effector function of antigen-specific T cells upon binding to ligands [82]. PD-1 inhibitors directly regulate the functions of various types of PD-1-expressing immune cells [83]. Immunotherapy with anti-PD-1 antibodies is widely used for the treatment of metastatic solid tumors, with a response rate of 20–55% [3]. Biomarkers to predict which patients are likely to respond to anti-PD-1 antibody therapy are needed.

Because highly variable CDR3 of the TCR chain is unique to individual T cell clones, CDR3 can be used to monitor the dynamics of T cell repertoire responses to ICIs [84]. A recent study showed that the TCR clonality or diversity of T cells in the blood increases 3 weeks after the initiation of treatment with anti-PD-1 antibodies [7]. Clonal proliferation of TCRs in the blood also occurred only in responders 3 weeks after the initiation of combination treatment with anti-PD-1 and anti-CTLA-4 antibodies. Thus, with this approach, minimally invasive liquid biopsies may be used in the early stages of treatment to predict patients’ treatment response.

The anti-PD-1 antibody treatment seems to be more effective in melanoma patients when the pretreatment TCR repertoire of TILs is larger in metastatic tumors [85]. An analysis of melanoma in The Cancer Genome Atlas reveals that a larger TCR repertoire of TILs is associated with longer overall survival even without anti-PD-1 antibody treatment. Furthermore, TCR repertoires in the peripheral blood of melanoma patients were examined to determine whether the TCR diversity predicted the clinical prognosis of ICI treatment [20]. Higher TCR repertoire diversity in the blood was associated with longer progression free survival, and low repertoire diversity was associated with poor prognosis. The diversity in patients who experienced late recurrence and long-term survival was significantly higher than that in rapid progressors. The TCR repertoire diversity in tumors may have a potential prognostic value.

Liquid biopsies were performed before treatment to predict responses to ICIs [86]. In the PCR analysis of pretreatment peripheral blood mononuclear cells (PBMCs), the diversity evenness of the TCRs repertoire score was correlated with the therapeutic efficacy of ICIs. Furthermore, the pretreatment level of TCR repertoire restrictions in CD4-positive T cells in peripheral blood had a potential prognostic value for clinical response to CTLA4 inhibition [20]. In addition, T cells release their DNA into the blood when cell death occurs. The released cell-free DNA in the blood was sequenced for analyzing CDR3 of T cells [63], which suggested that the clonal proliferation of T cell repertoire in the blood within 3 weeks of starting ICI treatment predicts the therapeutic efficacy.

### 4.6. Immune-Related Adverse Events and Repertoires

Anti-CTLA-4 and anti-PD-1 therapies can prolong survival in melanoma patients; however, these therapies can also induce organ-specific toxicities, called immune-related adverse events (IrAEs), which make it impossible to continue ICIs in a considerable number of patients [7,87]. It is likely that T cell clonal analysis is useful for the early diagnosis of IrAEs. The number of T cell clones that newly underwent proliferation was higher in patients with severe IrAEs after CTLA-4 blockade [88]. Furthermore, newly expanded clones were found among CD8-positive T cells, but not CD4-positive T cells, in patients with IrAEs [89]. In addition, severe repertoire restrictions were found in CD4-positive T cells in a study using samples of severe colitis [22]. CD4-positive and CD8-positive T cells may be diversely involved in various IrAEs; further studies are necessary for clarifying this point.

### 4.7. Repertoire Analysis and Vaccines

Peptides recognized by TCRs are used as vaccines to enhance antitumor immune responses [90]. Specifically, tumor biopsy specimens and nonmalignant tissue samples (usually PBMCs) are collected from patients and subjected to whole exome sequencing for comparison between tumor DNA and germline DNA to identify tumor-specific somatic mutations. A computational approach is used to predict MHC class I-binding epitopes, and peptides predicted to have moderate-to-high MHC-binding affinities are likely to induce CD8-positive T cell responses [91].

MHC class II-binding peptides are often more difficult to predict. The peptide-binding groove of MHC class I has closed ends to define the arrangement of a peptide epitope composed of 8–11 amino acid residues for presentation to CD8-positive T cells. In contrast, the peptide-binding groove of MHC class II has open ends and can bind to a peptide that is longer and more variable in length. Recently, new methods have been developed for the prediction of MHC class II-binding peptides [92,93,94], and they are expected to promote the development of MHC class II-binding peptides to elicit tumor-specific responses of CD4-positive T cells.

## 5. Repertoires and Tumor Heterogeneity

Relationships between TILs and melanoma during ICI therapy have been analyzed [95]. In responders to ICIs, the proportion of neoantigen-expressing tumors was reduced after the treatment, and tumor-infiltrating T cell clones expressing TCRs that recognize neoantigens were increased inversely. This result suggests that the proliferated neoantigen-recognizing T cell clones remove neoantigen-expressing tumor cells in responders to ICI therapy. Conversely, in nonresponders, clones with new mutations emerge during the treatment.

Repertoire analysis of CD8-positive T cells from tumors at different sites has also been performed [96]. Common CD8-positive T cell clones were found in tumors at different sites; however, the number of CD8-positive T cells for those clones varied widely depending on the tumor site. Because tumors at different sites express various tumor antigens in different ratios, CD8-positive T cell counts for clones should differ depending on the tumor site.

## 6. Symbiotic Microorganisms and Repertoires

### 6.1. Intestinal Bacteria

The compositions of intestinal bacteria in responders to ICIs are different from those in nonresponders [97,98]. Intestinal bacterial flora affects the innate and adaptive immune systems, thereby regulating the effectiveness of various antitumor treatments [99]. A recent study revealed how antigen mimicry between symbiotic bacteria and tumor antigens induces beneficial antitumor responses [100]. In a mouse model, *Bifidobacterium* spp., whose components have antigenic homologies to neoantigens of mouse melanoma, stimulate tumor-specific T cells in the form of cross-reactivity. *Bifidobacterium* spp. have also been shown to induce clonal proliferation of T cell repertoires that recognize neoantigens. In fact, fecal microbiota transplantation increased the tumor immunity [101]. Collectively, homologies between tumor neoantigens and pathogens or symbiotic bacteria may provide an effective method for isolating a unique T cell subpopulation with a potent antitumor function.

### 6.2. Intracellular Bacteria

Various bacteria are known to colonize human tumors [102]. Tumor-associated bacteria may affect immune function, treatment responses, and patient survival [103]. However, it remains to be fully elucidated whether antigens derived from intracellular bacteria are presented by MHC class I and II of tumor cells or whether such antigens induce TILs.

Recently, 16S rRNA gene sequencing was used to analyze intratumoral bacteria in melanoma [104], which found 41 bacterial species in 17 metastatic melanomas. High similarities in the composition of the identified bacteria were shown among samples from different patients, which suggested the presence of common bacterial species in melanoma cells. MHC peptidomics analyses have revealed 248 and 35 unique class I and class II peptides derived from 41 bacterial species. In addition, peptides derived from intracellular bacteria may stimulate CD8-positive T cells via MHC class I on tumor cells. Extracellular bacterial proteins taken up by tumors may stimulate CD4-positive T cells via MHC class II on tumor cells [105]. Bacterial antigens will be useful targets for immunotherapy as nonself antigens. In fact, TILs involved in the identification of bacteria-derived antigens were shown to secrete IFNγ and express CD69.

### 6.3. Viruses

CD8-positive T cells play a role in the detection and removal of cells presenting abnormal peptides on the surface as a result of viral infection or malignant transformation. Interestingly, antiviral T cells have been detected in the tumor microenvironment [106]. Tumor-specific peptides are occasionally similar to virus-derived peptides [107]. PBMCs were collected from melanoma patients before and after anti-PD-1 antibody treatment, and the cross-reactivity of PBMCs to tumors and cytomegalovirus (CMV) peptides was assessed. PBMCs were stimulated by both tumor peptides and CMV homologs, which suggests that CMV peptides expand virus-specific T cell clones capable of attacking and killing melanoma cells.

Meanwhile, CD8-positive memory T cells that do not recognize tumor antigens; however, these cells can recognize EB virus antigens, have been reported to account for 22% of TILs [108]. TILs recognizing tumor antigens can be distinguished from such non-tumor-reactive TILs based on higher levels of TCF7 transcription, PD-1 expression, and CD39 expression.

## 7. Summary

Analysis of immune cell repertoires has led to the elucidation of antitumor immune responses to melanoma. In particular, repertoire changes are important to predict the therapeutic efficacy of ICIs. The knowledge acquired through repertoire analysis of immune cells can be applied for enhancing tumor immune responses to improve the prognosis of melanoma. Currently, methods to enhance the therapeutic efficacy, such as the combined use of different ICIs, are being tested in many ongoing clinical trials. Repertoire analysis is expected to be performed in these trials. It is also important to study the effects of tumor heterogeneity and symbiotic bacteria (e.g., intestinal bacteria) on immune cell repertoires. Further studies are necessary to determine whether the results of immune cell repertoire analysis in melanoma can be extrapolated to other types of cancers.
